# Child drowning in northern Bangladesh: barriers, local initiatives, and opportunities for prevention

**DOI:** 10.3389/fpubh.2026.1849358

**Published:** 2026-09-23

**Authors:** Edris Alam, Ishrar Tahmin Anika, Jahangir Alam, Habiba Akter

**Affiliations:** 1 Rabdan Academy, Faculty of Resilience, Abu Dhabi, United Arab Emirates; 2 Department of Geography and Environmental Studies, University of Chittagong, Chittagong, Bangladesh; 3 Institute of Disaster Management and Vulnerability Studies, University of Dhaka, Dhaka, Bangladesh; 4 Department of Disaster Management, Begum Rokeya University, Rangpur, Bangladesh; 5 Disaster and Development Organization (DADO), Chattogram, Bangladesh

**Keywords:** Bangladesh, child safety, community-based approaches, death, drowning, prevention measures, security

## Abstract

**Background:**

Although Bangladesh has achieved substantial progress in reducing mortality from infectious diseases, child drowning, particularly in the northern regions, remains a neglected public health concern and constitutes the leading cause of death among children aged 1–17 years in rural areas. Approximately 18,268 children (aged 0–17 years) die from drowning annually, equivalent to an average of 50 deaths per day.

**Objective:**

This study aims to identify the socio-demographic characteristics of caregivers, the types of child supervision and reasons for its absence, water safety skills, traditional rescue practices, and the community’s preferences for child drowning prevention interventions in northern Bangladesh.

**Methods:**

We conducted face-to-face in-depth interviews and case-story analysis with 117 households that experienced drowning in two selected unions in northern Bangladesh. We employed quantitative analysis to examine the socio-demographic characteristics of the parents and carers, along with a mixed-methods approach for data collection on the reasons for a lack of adult supervision, water safety and rescue knowledge, the community’s role in preventing child drowning, and the assistance required from public and private institutions to prevent child drowning in the region.

**Results:**

After visiting a total of 18,004 households, we identified 117 drowning incidents, including 85 fatal and 32 non-fatal drownings in the last 6 years. The households that experienced drowning had an average monthly income of BDT 7,610, a lower level of education, and were primarily involved in farming and daily wage earnings. The study found that low understanding of child drowning risk, unawareness, and a lack of active adult supervision of children were associated with drowning deaths. Multivariable logistic regression identified greater distance from the drowning site, caregiver age, and household size as significant factors associated with fatal drowning, while each additional metre of distance from the drowning site increased the odds of fatal drowning. We also found limited knowledge about water safety and a lack of swimming and rescue skills as major factors in child drowning deaths. At the community level, participants preferred installing fencing along pond walkways and other reservoirs.

**Conclusion:**

In the absence of nationwide community-based drowning prevention programmes, this study recommends active community engagement to raise awareness, enhance understanding of child drowning risks, strengthen water safety, swimming, rescue, and cardiopulmonary resuscitation (CPR) skills, and reduce harmful post-rescue practices. Given the prevalence of low-income households, communities often lack the financial capacity to fence or fill unnecessary water reservoirs. Consequently, effective prevention of child drowning in northern Bangladesh will require coordinated efforts among individuals, households, communities, local councils, and civil administrations.

## 1 Introduction

Drowning is a significant cause of death worldwide, especially among children and young adults. Each year, over 300,000 people lose their lives to drowning, with 90 per cent of these fatalities occurring in low- and middle-income countries (1). Among children, drowning ranks as one of the top causes of fatality, placing fourth for those between 1 and 4 years old, and third for those aged 5–14. Research from Bangladesh, India, Vietnam, and China consistently identifies drowning as the leading cause of death for children in the 1–4-year age group (2–4). Alarmingly, child drowning deaths are disproportionately higher among the poorest and least-educated populations, regardless of a country’s economic development. This is especially true in rural areas near water bodies, where communities often lack the resources to mitigate water-related risks (5). Drowning deaths can be prevented through programmes and policies that target known risk factors. However, such initiatives are still in their early stages in many developing countries. Gaining a better understanding of the causes of drowning, as well as assessing community preferences and needs through local data collection or surveillance, could help develop effective measures to prevent child drowning (6).

The Bangladesh Health and Injury Survey (BHIS) conducted in 2003 identified drowning as the leading cause of death among children aged 1–17 years. The follow-up survey in 2016, which analysed data from 299,216 households, found that drowning had become the third leading cause of injury-related mortality, following suicide and traffic accidents (7). According to the 2023 survey, approximately 18,268 children (0–17 years) died from drowning each year, which is equivalent to 50 per day. In many developing countries, including Bangladesh, child drowning rates are disproportionately high due to socio-economic, behavioral, geographic, and environmental factors (8–10). Increased risk of drowning-related deaths is associated with male gender, children under 5 years old, maternal illiteracy, seasonality, and lower socio-economic status. Major contributing factors to child drowning deaths in Bangladesh include lack of supervision and inability to swim (11). Young children face drowning risks from various water sources, such as buckets, bathtubs, ponds, or pools. In Bangladesh, to reduce flooding risks, homes are often built on elevated land created by excavating the surrounding area, which can lead to uncovered pits near the homes. Tragically, many drowning incidents involving children under five occur in these ponds and ditches, especially within 20 m of homes, and particularly during morning hours (9 a.m.—1 p.m.) (2, 11, 12).

Community-led interventions are increasingly recognised as a viable approach to addressing child drowning in low- and middle-income countries (LMICs). Although drowning is a global public health concern, the burden is disproportionately concentrated in LMICs, where approximately 90% of drowning deaths occur (1). Bangladesh is among the most affected settings globally due to its extensive network of rivers, ponds, canals, and seasonal water bodies, combined with high population density and limited access to formal drowning prevention interventions (2, 13). While previous studies have documented the magnitude and determinants of child drowning in central and southern Bangladesh (5, 11, 12), less is known about the experiences, perceptions, and prevention needs of communities in northern Bangladesh. The northern region is characterised by comparatively lower socio-economic development and higher poverty levels than many other parts of the country (14, 15). Combined with the widespread presence of natural and artificial water bodies, these conditions may create unique vulnerabilities to child drowning and underscore the need for context-specific prevention strategies. These initiatives often rely on local resources and knowledge, leveraging social networks to implement strategies such as day care centres, swimming and rescue training, and parental education programmes (16, 17). In Bangladesh, community-based day care models have significantly reduced drowning rates by providing supervised environments for children during peak drowning hours (12).

Additionally, swimming and rescue skills training has demonstrated success in empowering communities to respond effectively to emergencies. These interventions, including swimming lessons, community-based day care, and parental education programmes, aim to leverage local knowledge, social networks, and resources to prevent drowning incidents (18). However, implementing community-led interventions is not without challenges. Existing research highlights barriers such as insufficient funding, cultural resistance, a lack of trained personnel, and difficulties in sustaining long-term community engagement (19). In Bangladesh, seasonal water bodies and socio-economic disparities further complicate the landscape, limiting the adaptability and scalability of interventions (13, 20). While these challenges are well documented, there is a lack of in-depth understanding of how community perspectives, socio-cultural contexts, and local dynamics shape the implementation of drowning prevention strategies.

Several studies have reported circumstances associated with child drowning and derived prevention measures from pilot interventions in the middle and southern regions of Bangladesh (11–13). It is essential to understand the specific community’s perception of child drowning risks and to identify the most feasible prevention measures based on the community’s characteristics and geographic settings (21). Limited studies have focused on the demographics and socio-economic characteristics of caregivers and the need for an assessment of child drowning prevention measures in northern Bangladesh. This region lags behind other parts of Bangladesh in socio-economic development (15). For instance, the per capita gross domestic product (GDP) growth rate in Rangpur, a region in northern Bangladesh, is 4.1%, which is notably lower than the rates in Dhaka and Chittagong of 6.7 and 5.5%, respectively, representing the central and southeastern regions of the country (14). This highlights the need for a context-specific approach to drowning prevention in Bangladesh, where diverse geographic and cultural settings demand tailored solutions. By focusing on the challenges and opportunities of community-led interventions, this research aims to uncover context-specific insights to inform the development of more effective and sustainable solutions for child drowning prevention in Bangladesh.

### 1.1 Aims and objectives

The study aimed to explore the circumstances, community perceptions, and prevention practices related to child drowning in selected rural communities of northern Bangladesh.

Specifically, the objectives are to

- Describe the circumstances surrounding child drowning incidents, including supervision, location, and timing.
- Identify factors associated with fatal child drowning.
- Explore the community’s water safety knowledge and perceptions of the causes and risk factors of child drowning.
- Describe post-rescue responses and practices following drowning incidents.
- Assess community awareness and acceptance of existing and proposed drowning prevention interventions.
- Explore community-recommended strategies for preventing child drowning in rural northern Bangladesh.

### 1.2 Theoretical framework of the study

This study was informed by the social–ecological model (SEM), which conceptualises health outcomes as the result of interactions between factors operating at multiple levels, including the individual, interpersonal, community, organisational, and policy environments (22, 23). The SEM has been widely applied in injury prevention and public health research because it facilitates the understanding of how behavioral, environmental, social, and structural factors collectively contribute to injury risk (24). In the context of childhood drowning, risk is not solely determined by individual caregiver behavior; it is also influenced by family supervision practices, community environments, social norms, access to prevention services, and broader policy support. The SEM therefore provided an appropriate framework for examining caregivers’ perceptions of drowning causes, rescue practices, and preferred prevention measures in northern Bangladesh.

## 2 Research methodology

### 2.1 Mixed-methods design

To achieve the main aim and objectives of the research, the study applied a mixed-methods approach (25), collecting data through closed-ended survey questions (quantitatively) and open-ended interview questions, case studies, and observations (qualitatively). Both methods have their limitations. However, mixed methods provide more robust results than relying solely on qualitative or quantitative approaches (26, 27). The study adopted the World Health Organization (WHO) definition of drowning as “the process of experiencing respiratory impairment from submersion or immersion in liquid” (28). Drowning outcomes were classified as fatal drowning (death resulting from the drowning event) and non-fatal drowning (survival following a drowning event, regardless of subsequent health consequences). This definition was used during household screening and data collection.

This study adopted a convergent parallel mixed-methods design, in which quantitative and qualitative data were collected during the same field period and analysed separately before being integrated at the interpretation stage. Primary data were collected directly from participants using a convergent mixed-methods design. The primary dataset comprised two complementary components: (1) quantitative data obtained through a structured household questionnaire administered to all identified drowning-affected households, and (2) qualitative data collected through in-depth interviews with purposively selected caregivers to explore their experiences, perceptions, and recommendations regarding childhood drowning. The quantitative component focused on describing the magnitude and distribution of drowning incidents and the associated socio-demographic characteristics of affected households. In contrast, the qualitative component explored contextual explanations related to supervision practices, caregiver perceptions, traditional rescue behaviors, and community preferences for prevention strategies. Integration of the two strands occurred primarily through triangulation during the interpretation phase. Quantitative findings (e.g., patterns of supervision, socio-economic status, and prevalence of unsafe rescue practices) were systematically compared with qualitative narratives derived from interviews and case stories. This allowed for the examination of convergence and divergence between numerical trends and lived experiences. The integration revealed strong convergence between the quantitative and qualitative findings. The mixed-methods design provided insights that neither quantitative nor qualitative data could fully explain in isolation. While the quantitative results identified patterns in risk distribution and behavior prevalence, the qualitative data explained the underlying social, cultural, and contextual mechanisms driving these patterns (29).

### 2.2 Study area

Primary data were collected through interviews, case studies, and observations in two sub-districts, Debiganj and Taraganj, in the Rangpur division of Bangladesh. To minimise selection bias, one union from each sub-district was selected using simple random sampling from a complete list of unions obtained from local administrative records. Randomisation was performed using a computer-generated random number method. These upazilas were considered broadly representative of many rural communities in northern Bangladesh in terms of livelihood patterns, socio-economic conditions, and exposure to open water bodies, which are recognised risk factors for childhood drowning. Primary data were collected in Debiganj Upazila, Panchagarh District, and Taraganj Upazila, Rangpur District, both located in northern Bangladesh. The study sites were selected purposively based on three considerations: (i) documented occurrences of child drowning incidents in rural settings, (ii) representation of typical socio-economically disadvantaged communities in northern Bangladesh, and (iii) logistical feasibility for conducting a complete household-based survey. The availability of trained local surveyors was considered necessary to ensure comprehensive household coverage, data quality, and participant access but was not used as an indicator of drowning risk.

Within the selected upazilas, one union from each upazila (Chilahati and Hariarkuti) was randomly selected for field investigation. Debiganj is a sub-district in Panchagarh district comprising 10 unions. Chilahati Union is the lower-level local government unit divided into 9 wards. Of the 10 unions in this sub-district, Chilahati Union was randomly selected for the field survey. The total number of households in the union is 5,935 (30). Taraganj is a sub-district in Rangpur district comprising 5 unions. Of the five unions in this sub-district, Hariarkuti Union was randomly selected for the field survey. The total number of households in the union is 9,779 (31). A formal sample size calculation was not performed because the study adopted a census approach rather than a sample-based design. All households within the selected unions were visited during the household census, covering a total of 18,004 households, to identify every eligible household that had experienced a fatal or non-fatal drowning event during the 6-year recall period. Consequently, the quantitative sample comprised all eligible drowning-affected households identified through this census.

### 2.3 Data collection tools

We developed a baseline dataset on drowning incidents, the socio-demographic profiles of parents and caregivers, traditional practices for treating drowning victims, and community-based prevention measures over the 3 months from January to March 2024. Participants were asked to recall drowning incidents occurring in their households over an approximate 6-year period. Based on the reported responses, incidents were mainly distributed between 2020 and 2023, with a smaller proportion reported before 2020. Due to recall aggregation in earlier years, precise year-by-year classification was not available for all cases. Therefore, the study period is interpreted as approximately 2018–2023, depending on respondent recall.

The database and interview question format were developed by adapting components of the child drowning risk estimation index developed by Borse et al. (32), the water incidence index by Hills et al. (33), and the guidelines for community surveys on injuries and violence by WHO (34). Both quantitative and qualitative methods were applied to document and analyse drowning occurrences, associated risk factors, existing practices following drowning events, and potential drowning prevention measures in the study region. A pretested database format and interview tool were developed, incorporating structured, semi-structured, and open-ended questions. All data were collected through face-to-face interviews with the participants. The interview tool consisted of integrated sections combining both quantitative and qualitative components. The quantitative questions captured the socio-economic and demographic characteristics of households and drowning victims, including the age and sex of the child, the mother’s age, the occupation of the household head, and monthly household income. The qualitative questions explored contextual factors related to child drowning, including child supervision practices, circumstances surrounding drowning events, actions taken following rescue attempts, reasons for inadequate adult supervision, types of caregiver-related risks, swimming learning practices and locations, community perceptions and roles in drowning prevention, preferences for prevention interventions, and potential contributions of public and private institutions to reduce child drowning. The tool also included quantitative questions assessing swimming and water safety skills, knowledge of safe rescue procedures, CPR awareness, and willingness to participate in drowning prevention interventions. Participants’ perceived knowledge regarding water safety and child drowning prevention was measured using a five-point Likert scale (1 = Not knowledgeable at all, 2 = Little knowledgeable, 3 = Moderately knowledgeable, 4 = Knowledgeable, and 5 = Very knowledgeable).

### 2.4 Data collection process

To identify households that experienced a child drowning, data collectors visited every household in the allocated ward; they stopped and interviewed only if the drowning incident was either fatal or non-fatal and occurred in the last 6 years. If a child dies at any time because of drowning, this is termed a fatal drowning. We adopted the definition of non-fatal drowning provided by Rahman et al. (13), who defined it as: “*non-fatal drowning was operationally defined as survival from a drowning event*”. Thus, a total of 117 households were identified that faced fatal and non-fatal drowning in the last 6 years. These households provided contextual information on each drowning, rescue and treatment measures taken, and their preferences and expectations regarding child drowning prevention in the region. All questionnaires were written in Bengali, and a dedicated native Bengali-speaking person interviewed the participants. The proportion of missing data across variables was minimal (generally <2%), so it was unlikely to materially affect the findings.

Eighteen data collectors, one for each ward in a union, were employed to collect data, and two supervisors, one for each union, were employed to oversee and monitor the data collection process. All selected data collectors and supervisors were trained on the study objectives, interview questions, and ethics protocol. While visiting households, only family members who were available and comfortable answering questions were interviewed. A household member was defined as someone living in the same household, including domestic helpers or long-term guests who shared daily meals and participated in household activities. Repeat visits were made to households where any respondents were unavailable during the first visit. All interviews were recorded with participants’ prior verbal informed consent to ensure accuracy and transparency. The excerpts of the interviews were translated and transcribed into English by a certified translator who is also a researcher with bilingual expertise. Two authors independently reviewed each interview transcript to minimise bias and enhance reliability. Additionally, quality control measures were implemented, including cross-checking transcripts against recordings and conducting regular team discussions to resolve discrepancies and ensure consistency in data interpretation. These steps were taken to uphold the rigour and credibility of the research process.

### 2.5 Data analysis

Drowning incidence data collected through household visits and interviews were analysed to estimate drowning events over the preceding 6 years. The reported drowning cases were categorised by sex, age group, and location of occurrence. Socio-demographic characteristics of parents and caregivers were summarised using descriptive statistics. Categorical variables were compared between fatal and non-fatal drowning groups using Pearson’s chi-square test. Fisher’s exact test or Fisher–Freeman–Halton exact test was applied when the assumptions of the chi-square test were not met due to small expected cell frequencies. Continuous variables were assessed for normality using the Shapiro–Wilk test. Normally distributed variables were compared using the independent samples *t*-test, whereas non-normally distributed variables were analysed using the Mann–Whitney *U* test. Statistical significance was considered at *p* < 0.05.

Responses to Likert-scale questions were summarised using descriptive statistics, and mean scores were calculated for each item across participants. Open-ended and semi-structured responses were analysed thematically by organising the findings according to key themes, including types of adult supervision, rescue and treatment practices following drowning incidents, community acceptance and participation in drowning prevention measures, and perceived support required from public and private sectors. A multivariable binary logistic regression analysis was conducted to identify factors independently associated with fatal drowning. Variables with *p* < 0.20 in the bivariate analysis, along with variables considered epidemiologically relevant based on previous literature and biological plausibility, were included in the regression model. For the multivariable logistic regression analysis, selected categorical variables were collapsed into fewer categories to ensure adequate cell counts, improve model stability, and avoid sparse data bias. Adjusted odds ratios (AORs) with 95% confidence intervals (CIs) were reported. To improve model stability, categories with sparse observations were combined before conducting logistic regression while maintaining meaningful epidemiological interpretations. Model adequacy was assessed using appropriate goodness-of-fit statistics.

The responses to open-ended interviews were processed and analysed using NVivo 12. The analysis followed an inductive content analysis approach. Initially, interview transcripts were read repeatedly to achieve data familiarisation. Meaningful text segments relevant to drowning experiences, supervision practices, rescue measures, and prevention strategies were assigned initial codes. These codes were developed directly from the data rather than from a predetermined theoretical framework. Similar codes were subsequently grouped into categories and then synthesised into broader themes through an iterative review process. Emerging themes were continuously refined through comparison across transcripts until conceptual consistency was achieved. Two members of the research team were involved in the coding process. An initial coding framework was developed by the lead analyst and independently reviewed by a second researcher. Coding discrepancies were discussed and resolved through consensus meetings until agreement was reached. Formal inter-rater reliability statistics were not calculated because the purpose of the analysis was interpretive rather than measurement-oriented.

### 2.6 Ethical considerations

All participants were informed of the research aims and objectives. In the absence of literacy among many participants, verbal informed consent was sought from all the participants who took part in the interviews. They were given the option to withdraw from interviews at any stage. No names or identifiable details of the participants were used, including their institution and position, for anonymity purposes. The Rabdan Academy has approved human subject consultation for this study (#RAREC00040) and the Ethics Committee of the University of Chittagong, Chittagong 4,331, Bangladesh (CUNI # 0180).

## 3 Results

### 3.1 Socio-demographic characteristics of the respondents

By visiting 18,004 households, we found 117 drowning incidents in the Chilahati and Hariarkuti unions over the last 6 years. Table 1 presents the household and caregiver characteristics of the study participants (*n* = 117). 62.4% of the respondents were male and the rest were female. Most children belonged to nuclear (living only with parents and children) families (79.5%), and most caregivers were 22–29 years of age (45.3%). Farming was the predominant occupation of the household head (43.6%), while nearly two-thirds of households (65.8%) reported a monthly income of BDT 5,001–10,000. The house and adjoining homestead, often called a *bari,* where approximately 99 per cent *of baris* have more than one household. Almost half of the households (45.3%) had two houses within the *bari.*

**Table 1:** Demographics and socio-economic characteristics of the respondents.

Items/characteristics	Frequency (*n* = 117)	Percentage
Gender		
Male	73	62.4
Female	44	37.6
Age (years)		
Below 18	4	3.42
18–21	34	29.06
22–29	53	45.30
30–39	24	20.51
40–49	2	1.71
Family type		
Nuclear family	93	79.5
Joint family	24	20.5
Number of family members		
1–4	50	42.7
5–6	47	40.2
7 or more	2	17.95
Major occupation of the household head		
Farmer	51	43.59
Day labourer	27	23.08
Small traders	22	18.80
Fisherman	17	14.53
Monthly income		
1–5,000	12	10.3
5,001–10,000	77	65.8
10,001–15,000	16	13.7
15,001–20,000	12	10.26
Number of houses in a *bari*		
1	1	0.9
2	53	45.3
3	45	38.5
4	10	8.5
4 and above	8	6.8

Source: authors.

### 3.2 Circumstances surrounding child drowning incidents

Among the 117 drowning cases, 85 (72.7%) were fatal and 32 (27.3%) were non-fatal (Table 2). Children aged 1–4 years accounted for the majority of both fatal (64.7%) and non-fatal (62.5%) drowning cases, and boys predominated in both groups (74.1 and 81.3%, respectively). Fatal drownings occurred predominantly in canals, rivers, and other natural water bodies (72.9%), whereas ponds accounted for most non-fatal drowning incidents (75.0%). Most drowning events occurred between 11:00 and 14:00 h, with the highest frequencies recorded between 11:00 and 12:00 (42.4% of fatal and 56.3% of non-fatal cases). Drowning incidents were most common during the monsoon months, particularly July and September. The distribution of drowning outcomes varied significantly across months, with fatal cases showing a higher occurrence in September, whereas non-fatal cases were more frequent in July. Fatal drowning cases occurred at a greater mean distance from households compared with non-fatal cases (41.79 m vs. 26.97 m). Approximately three-quarters of children were not under adult supervision immediately before the drowning event in both fatal (72.9%) and non-fatal (68.8%) cases. Most affected households belonged to nuclear families, and caregivers were predominantly aged 22–29 years. Most fatal (69.4%) and non-fatal (56.3%) drowning cases occurred among households with a monthly income of BDT 5,001–10,000. Low parental educational attainment was common in both groups, with 44.7% of mothers and 41.2% of fathers of fatal cases, and 59.4% of mothers and 56.3% of fathers of non-fatal cases having no formal education.

**Table 2:** Distribution of characteristics of drowned persons and drowning circumstances by drowning outcome (fatal and non-fatal cases).

Category	Fatal	Non-fatal	*p*-value
Total cases	85 (72.65)	32 (27.35)	
Age (years)			0.247
1–4	55 (64.7)	20 (62.5)	
5–8	24 (28.24)	12 (37.5)	
9 and above	6 (7.06)	–	
Gender			0.420
Male	63 (74.1)	26 (81.3)	
Female	22 (25.9)	6 (18.8)	
Drowning location			0.363
Pond	17 (20.0)	24 (75.0)	
Canals, rivers, seas	62 (72.9)	8 (25.0)	
Sink	6 (7.1)	–	
Depth of drowning site (feet)	6.34 ft	7.875 ft	0.559
Distance from drowning site (m)	41.79 m	26.9688 m	0.017
Drowning time			0.647
0–8	1 (1.2)	–	
9–10	15 (17.6)	2 (6.3)	
11–12	36 (42.4)	18 (56.3)	
13–14	19 (22.4)	7 (21.9)	
15–16	10 (11.8)	4 (12.5)	
17–18	4 (4.7)	1 (3.10)	
Drowning month			0.045
April	1 (1.2)	–	
May	3 (3.5)	3 (9.4)	
June	13 (15.3)	8 (25.0)	
July	16 (18.8)	11 (34.4)	
August	6 (7.1)	5 (15.6)	
September	23 (27.1)	2 (6.3)	
October	11(12.9)	1 (3.1)	
November	2 (2.4)	1 (3.1)	
December	7 (8.2)	1 (3.1)	
Drowning year			0.253
Before 2020	24 (28.2)	9 (28.1)	
2020	7 (8.2)	6 (18.8)	
2021	17 (20.0)	2 (6.3)	
2022	12 (14.1)	6 (18.8)	
2023	25 (28.2)	9 (28.1)	
Learnt swimming			
Yes			
Supervision status			0.653
Yes	23 (27.1)	10 (31.3)	
No	62 (72.9)	22 (68.8)	
Caregiver’s age			0.086
Below 18	3 (3.5)	1 (3.1)	
18–21	28 (32.9)	6 (18.8)	
22–29	41 (48.2)	13 (40.6)	
30–39	12 (14.1)	11 (34.4)	
40–49	1 (1.2)	1 (3.1)	
Occupation of the household head			0.202
Day labourer	22 (25.9)	5 (15.6)	
Farmer	32 (37.6)	19 (59.4)	
Fisherman	14 (16.5)	3 (9.4)	
Small businessman	17 (20.0)	5 (15.6)	
Family members of the drowned person			0.282
1–4	33 (38.8)	17 (53.1)	
5–6	37 (43.5)	9 (28.1)	
7 or more	15 (17.6)	6 (18.8)	
Family type			0.422
Nuclear family	66 (77.6)	27 (84.4)	
Joint family	19 (22.4)	5 (15.6)	
Monthly household income (BDT)			0.022
1–5,000 Tk	4 (4.7)	8 (25.0)	
5,001–10,000 Tk	59 (69.4)	18 (56.3)	
10,001–15,000 Tk	12 (14.1)	4 (12.5)	
15,001–20,000 Tk	10 (11.8)	2 (6.3)	
Maternal education			0.279
No formal education	38 (44.7)	19 (59.4)	
Primary	23 (27.1)	8 (25.0)	
Secondary	24 (28.2)	5 (15.6)	
Paternal education			0.410
No formal education	35 (41.2)	18 (56.3)	
Primary	28 (32.9)	8 (25.0)	
Secondary	13 (15.3)	5 (15.6)	
Graduate	9 (10.6)	1(3.1)	

Source: authors.

Fisher’s exact test was used where expected cell counts were sparse (>20% of cells had expected frequencies <5) and the significance level, *p* ≤ 0.05.

Among the examined socio-demographic and environmental characteristics, significant associations with drowning outcomes were observed for distance from the drowning site (*p* = 0.017), drowning month (*p* = 0.045), and household income (*p* = 0.022). No statistically significant associations were found for age, sex, drowning location, drowning time, drowning year, supervision status, caregiver age, occupation, family characteristics, or parental education (*p* > 0.05).

In response to questions about caregivers’ activities during child drowning, four types of responses were recorded. Out of 117 households, in approximately 90 per cent of cases, participants frequently reported that caregivers, particularly mothers, were engaged in household chores at the time of the drowning incidents. Several interview participants described these activities as the circumstances surrounding the incidents. The remaining three types of activities, including working in the field, chatting with someone, and going elsewhere, accounted for 6, 2.6, and 1.7 per cent, respectively. In the village of Char Tistapara, located in Debiganj sub-district, tragedy struck when a 2-year-old boy drowned. When questioned about the incident, the mother of the deceased child shared her heart-breaking account in Bengali, which translated as follows:

*I can hardly find words to express the sorrow and pain of this tragic death. I had a 2-year-old son, who was dearly loved and emotionally connected to me. One day, I went about my usual task of tending to our goats, unaware that my son had quietly followed me. I failed to notice him trailing behind me. Lost in my chores, I spent considerable time in the fields. Upon my return, I realised he was missing. Panic ensued as we searched frantically, only to find him floating lifelessly in the nearby pond. Despite immediate rescue efforts and attempts to revive him, it was too late. Oh, I wish I had known he was following me! Perhaps then, this tragedy could have been averted. But alas, it seems our fate was written in sorrow.*

### 3.3 Multivariable analysis of factors associated with fatal drowning

Variables significant in bivariate analyses were subsequently examined in multivariable logistic regression. Multivariable binary logistic regression analysis identified distance from the drowning site, caregiver age, and number of family members as significant predictors of fatal drowning (Table 3). After adjusting for other covariates, each 1-metre increase in the distance between the household and the drowning site increased the odds of fatal drowning by 3.1% (AOR = 1.03, 95% CI: 1.00–1.06, *p* = 0.026). Compared with caregivers aged <22 years, drowning cases involving caregivers aged ≥30 years had significantly lower odds of fatal drowning (AOR = 0.28, 95% CI: 0.08–0.96, *p* = 0.042). Similarly, cases from households with five or more family members had lower odds of fatal drowning compared with those from households with 1–4 members (AOR = 0.34, 95% CI: 0.12–0.98, *p* = 0.045).

**Table 3:** Multivariable binary logistic regression analysis of factors associated with fatal drowning.

Category	AOR (95%CI)	*p*-value
Age		
1–4	Ref.	–
5 and above	1.55 (0.54–4.45)	0.415
Gender		
Male	Ref.	–
Female	0.41 (0.12–1.47)	0.172
Distance from drowning site (m)	1.03 (1.00–1.06)	0.026*
Drowning month		
Monsoon	Ref.	–
Non-monsoon	0.60 (0.19–1.91)	0.391
Supervision status		
Yes	Ref.	–
No	3.31 (0.85–12.80)	0.084
Caregiver’s age		
<22	Ref.	–
22–29	2.59 (0.66–10.22)	0.174
≥30	0.28 (0.08–0.96)	0.042*
Family members of the drowned person		
1–4	Ref.	–
5 or more	0.34 (0.12–0.98)	0.045*
Monthly household income (BDT)		
≤10,000	Ref.	–
>10,000	1.19 (0.36–3.96)	0.779
Maternal education		
No formal education	Ref.	–
Educated	1.27 (0.28–5.66)	0.755
Paternal education		
No formal education	Ref.	–
Educated	1.37 (0.32–5.93)	0.677
Hosmer–Lemeshow goodness-of-fit test	0.317	
Cox and Snell *R* square	0.193	
Nagelkerke *R* square	0.279	

Source: authors.

AOR, adjusted odds ratio; CI, confidence interval and significance level, *p* ≤ 0.05*.

Other factors, including the age of the drowned person, gender, drowning month, supervision status, household income, maternal education, and paternal education, were not statistically significantly associated with fatal drowning after adjustment. Although supervision status showed increased odds of fatal drowning among unsupervised cases (AOR = 3.31, 95% CI: 0.85–12.80), the association did not reach statistical significance (*p* = 0.084). The final model demonstrated acceptable goodness of fit (Hosmer–Lemeshow test *p* = 0.317) and explained 27.9% of the variance in fatal drowning based on Nagelkerke *R*^2^.

### 3.4 Community knowledge and perceptions of drowning risk

Out of 117 households, mothers were the primary caregivers for 78.6 per cent of children, whereas grandparents and others accounted for 19.7 and 1.7 per cent, respectively (Table 4). Thirty-six (30.8%) reported that their child had been taught swimming skills, whereas 81 (69.2%) reported that their child had not been taught to swim. Among those who had received swimming instruction (*n* = 36), the majority learned swimming by themselves (52.8%), followed by learning from parents (38.9%) and grandparents (8.3%). Regarding knowledge of safe rescue procedures, only two respondents (1.7%) reported knowing safe rescue procedures, while the vast majority (98.3%) lacked knowledge of safe rescue practices. Furthermore, none of the respondents (100%) had taught their child what to do if someone was drowning. Regarding perceptions of child drowning, most respondents (80.3%) considered parental or caregiver negligence to be responsible for child drowning in their area. As a preferred preventive measure, the vast majority (89.7%) suggested installing barriers at the entrance of water reservoirs, whereas only a small proportion recommended establishing childcare facilities (6.0%) or conducting awareness activities (4.3%).

**Table 4:** Water safety knowledge and perceptions among drowning-affected households (*n* = 117).

Question	Frequency	Percentage
Primary caregiver of the drowned children		
Mother	92	78.6
Grandparents	23	19.7
Others	2	1.7
Child taught to swim		
Yes	36	30.8
No	81	69.2
Person who taught swimming (*n* = 36)		
Self-taught	19	52.8
Parent	14	38.9
Grandparent	3	8.3
Knowledge of safe rescue		
Knew safe rescue procedures	2	1.7
Did not know safe rescue procedures	115	98.3
Taught their child what to do if someone was drowning		
Yes	0	0.0
No	117	100.0
Do you agree that parental/caregiver negligence is responsible for child drowning in your area?		
Yes	94	80.3
No	23	19.7
What would you like to do as a personal initiative to prevent child drowning in your area?		
Conducting awareness activities	5	4.3
Establish childcare	7	6.0
Installation of barriers at the entrance of reservoirs	105	89.7

Among all the participating households, 108 (92.3%) stated one or more reasons for inadequate child supervision linked to drowning (Table 5). Several participants described these events as reflecting shortcomings in child supervision. The most frequently cited reasons were parental unawareness, negligence, or a low perception of drowning risk (76 households, 65.0%), followed by caregivers being occupied with household work, particularly mothers (54 households, 46.2%).

**Table 5:** The reasons for lack of adult supervision and types of caregiver negligence.

Types of negligence	Responses (*n* = 148)	% of responses	% of households
Unawareness/negligence/allowing child to play alone	76	51.4	65.0
Mother busy with household chores	54	36.5	46.2
Accident/unavoidable event	12	8.1	10.3
Poverty	4	2.7	3.4
*Jinn*/ghost influence	2	1.4	1.7

Multiple responses were counted (source: authors).

Quantitative findings indicated that nearly three-quarters of children were reportedly unsupervised immediately before the drowning incident. Qualitative interviews provided additional context, with caregivers describing household responsibilities and the absence of alternative childcare arrangements as reasons why children were left unattended. As one mother explained:

*I was sitting in the yard of my house with the child. Then I left him sitting there and went to cook. I became busy preparing food for family members and left the child unattended for a while. After an hour, I searched for the child and later saw the child floating in the nearby pond.*

Similar accounts were reported by several caregivers, who described balancing household responsibilities while supervising young children. By viewing this as a failure in child protection, the participants stated that if parents had taken care of the child, he would not have fallen into the water. A participant (55) from Balrampur, Chilahati Union in Debiganj reported:

*We did not take care of the child for a long time, which is why he was left alone. We also let the child go outside alone.*

Parental busyness at work, particularly mothers being occupied with household chores, was mentioned as the second most common reason for child drowning deaths. Caregivers frequently reported that they suddenly could not find their child and began searching for them. A relevant scenario comes from a mother (35) in Chilahati Union, Debiganj, who lost her 2-year-old child to drowning:

*I was sitting in the yard of my house with the child. Then I left him sitting there and went to cook. I became busy preparing food for family members and left the child unattended for a while. After an hour, I saw the child in the courtyard and searched for a long time. Later, I saw the child floating in the nearby pond.*

Participants overwhelmingly perceived inadequate supervision as the most important circumstance surrounding child drowning incidents. Caregivers frequently described situations in which children were left unattended while adults worked in the household or on the farm. Participants also described household labour responsibilities and the absence of alternative childcare arrangements as challenges to maintaining continuous supervision. Several participants interpreted these incidents as preventable and expressed regret regarding supervision practices. However, many also emphasised that continuous supervision was difficult because household labour responsibilities often fell on mothers.

Playing is the most frequently reported activity before a child drowns. Caregivers often leave children to play with peers for extended periods without any adult supervision while they engage in various activities. Children were often found playing on the banks of ponds, rivers, and canals, and were sometimes allowed to play and bathe in open water alone. A father (45) from Sheikh Badha in Chilahati Union, Debiganj, who lost his 2-year-old son to drowning, stated, *for a long time, none of us paid attention to where the boy went; we were just doing housework*.

Around 8 per cent of the participants claim they took care of their children adequately, and that it happened by accident. This group of participants argues that parents did not want the child to die on purpose; therefore, they are not responsible for it. A resident (45) of Kajaldighi, Chilahati Union, in Debiganj claimed that it was an accident in which no one had a hand. A father (35) from Chilahati in Debiganj asserted that:

*We always take care of the child. The child was playing in the courtyard while his mother was working. However, in between, the child went to the pond and drowned. We did not have a second person to always care for the child. I cannot accept it entirely as parental negligence, because no parent wants their child to die.*

A striking sociological finding that emerged from the data is that respondents attributed 3 of 117 drowning incidents (2.56%) to supernatural causes, specifically jinn or ghosts. These cases were reported exclusively from the Debiganj area of Chilahati Union. In one non-fatal case involving a 2-year-old girl, the respondent believed a *jinn* had thrown the child into the water; the child remained unconscious for hours but recovered after treatment by a traditional healer called *Kabiraj*. In a fatal case of a 5-year-old boy, the mother attributed the death to a jinn bite, citing as evidence that the child’s body was found standing upright in the pond rather than floating. In a third fatal case involving an 11-year-old madrasa student who knew how to swim, the respondent believed a jinn killed the child because he ‘knew Satan.’ Notably, in two of the three cases, respondents explicitly rejected parental negligence as an explanation.

### 3.5 Post-rescue responses and practices

Overall, participants reported limited knowledge of water safety, drowning prevention, and rescue practices. Their self-reported knowledge levels are presented in Figure 1 and were further illustrated during qualitative interviews. None of the households considered themselves either knowledgeable or very knowledgeable. A vast majority, 44.4 per cent of households, considered themselves not knowledgeable at all. Little knowledge and moderately knowledgeable comprised 49.6 and 6 per cent, respectively.

**Figure 1:**
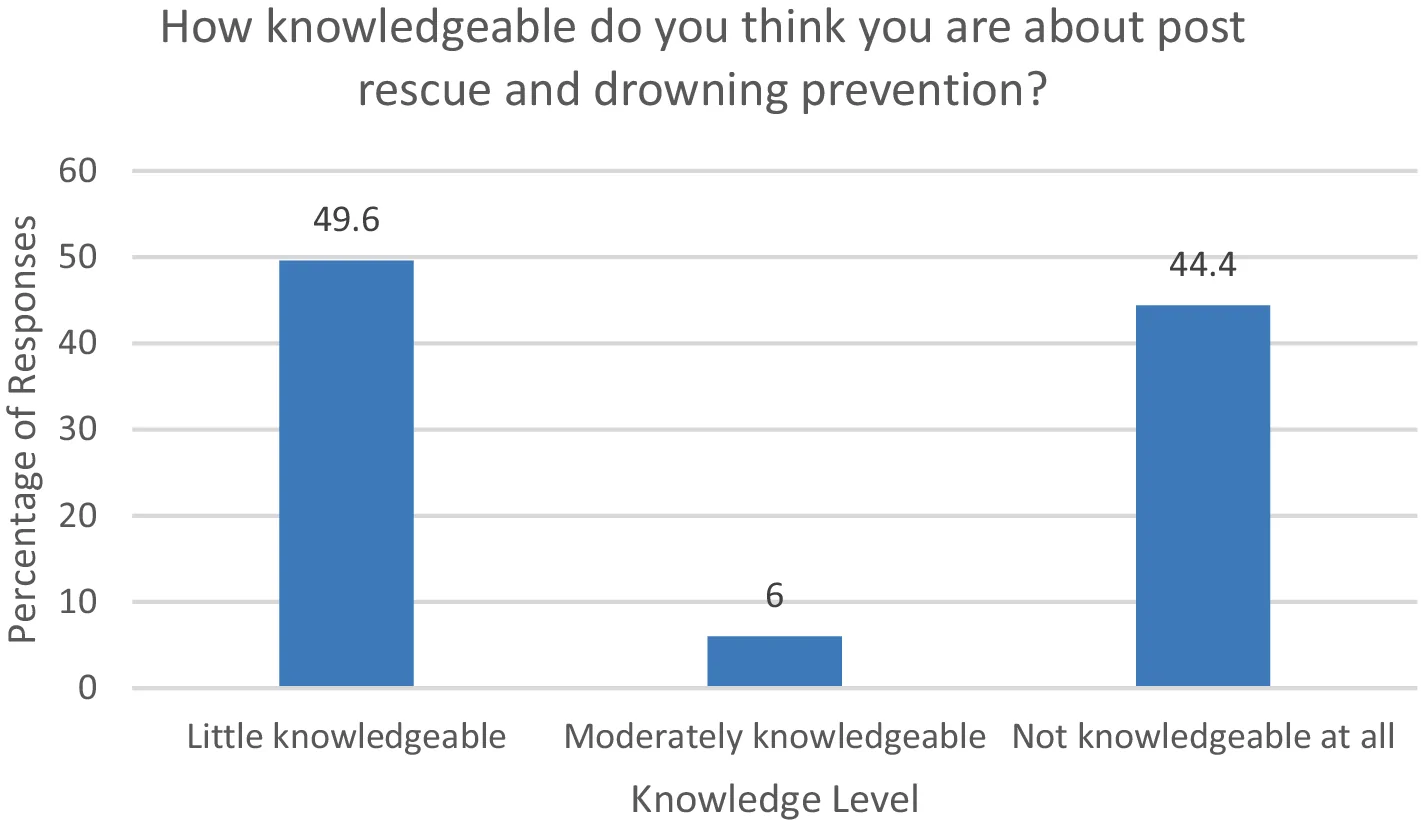
Self-reported knowledge regarding childhood drowning prevention among drowning-affected households (*n* = 117) (source: authors).

Post-rescue management was predominantly characterised by traditional practices rather than evidence-based first aid. The most frequently reported response was abdominal pressure combined with spinning the child over the shoulder (24.8%), followed by massaging with oil and salt while spinning the child (9.4%) and rotating the child over the shoulders or head (8.6%) (Table 6). Only 6.8% of participants reported performing CPR, while an equal proportion attempted to expel water by pressing the child’s stomach. Direct transfer to a hospital was reported by 8.6% of respondents. Notably, 24.8% of participants could not recall the measures taken following rescue, highlighting limited awareness and inconsistent post-rescue practices among caregivers.

**Table 6:** Treatment for drowned child after rescue.

Treatment for drowned child after rescue	Frequency (*n* = 117)	Percentage
1. Perform CPR	8	6.8
2. Abdominal pressure and spinning the child over the shoulder	29	24.79
3. Massaging with oil and salt and spinning the child over the shoulder	11	9.40
4. Rotating by taking child over the shoulders or head	10	8.55
5. Taken directly to hospital	10	8.55
6. Water is extracted by pressing the stomach	8	6.84
7. Discharged water by pressing the stomach and taken to the doctor	8	6.84
8. Rotating over shoulders and taken to hospital	7	5.98
9. Trying to expel water from the stomach by pressing from the back	3	2.56
10. Massaging hands and feet with oil and seeking a doctor	2	1.71
11. Do not remember	29	24.78
Total	117	100

Source: authors.

On occasions, a child who was rescued survived but later died while the rescuer was executing malpractice during treatment processes. For example, a 5-year-old child at Mahatpara in Debiganj was rescued and survived but died while being spun over the rescuer’s shoulder. Similarly, another 3-year-old child was rescued and survived but died while abdominal pressure was applied by supination along with spinning the child over the shoulders at Chochpara in Chilahati in Debiganj. We counted a total of seven cases where children who were rescued survived but died while receiving treatment in the post-rescue period. The third most reported malpractice involved rotating the child on the shoulders or head.

Several participants linked multiple child drowning incidents to limited awareness regarding safe rescue. A father (34) from Sarkarpara of Chilahati Union at Debiganj stated,

*Our two sons were playing on the riverbank. The younger son went down into the water. But at one point, he started to get swept away by the river. The elder son saw it and jumped into the river, and the two hugged. Thus, they drowned to death together.*

A small but important subset of cases involved children who were reportedly rescued alive but subsequently died after traditional post-rescue practices were applied. Although caregivers perceived these practices as part of the treatment process, the present study cannot determine whether they influenced mortality. Out of the total 85 fatal drowning cases, seven cases involved children who initially survived immersion and were rescued alive but subsequently died during or after the application of harmful traditional treatment practices (Table 7). These cases were characterised by non-evidence-based interventions, including abdominal pressure, forceful inversion, and repeated body manipulation, which are commonly believed within the community to remove “water from the stomach.” In all seven cases, caregivers reported initial signs of recovery, such as breathing or movement, but deterioration occurred shortly after the application of these practices.

**Table 7:** Characteristics of post-rescue malpractice cases leading to secondary death (*n* = 7).

Age	Initial condition after rescue	Harmful practice applied	Time to death after rescue	Care-seeking behavior
2 years	Semi-conscious	Abdominal pressure	Shortly after	No formal care
3 years	Breathing	Abdominal pressure + inversion	Within hours	No hospital visit
	Breathing restored	Shoulder rotation	Same day	No hospital visit
4 years	Stable initially	Traditional massage + inversion	Within hours	Local healer
5 years	Conscious	Spinning over shoulder	Same day	Delayed care
	Responsive	Abdominal pressure + back pressing	Hours later	Delayed hospital
7 years	Initially survived	Combined multiple practices	Within hours	No medical care

### 3.6 Community awareness of drowning risk

The participants provided a total of 201 responses identifying the roles of neighbors and communities in preventing child drowning (Table 8). Regarding community-suggested strategies for preventing child drowning, the most frequently recommended measures were installing fencing or barriers around ponds and reservoirs (36.8%) and improving self-awareness and community awareness to ensure more active child supervision (36.3%). Less commonly reported strategies included teaching children to swim (9.5%), establishing childcare facilities (7.5%), and filling ponds, canals, or other nearby water bodies (6.5%). Only a small proportion of participants suggested preventing the construction of new reservoirs adjacent to houses (2.5%) or learning CPR skills (1.0%).

**Table 8:** Roles the neighbourhood or community can play to prevent child drowning.

Responses	Frequency (*n* = 201)	% of responses	% of households
1. Fencing/barrier around the pond/reservoir	74	36.82	63.25
2. Self-awareness and community awareness for more active child supervision	73	36.32	62.39
3. Teaching children to swim	19	9.45	16.24
4. Establishing childcare	15	7.46	12.82
5. Filling ponds/sinks/canals	13	6.47	11.11
6. Stopping the construction of new reservoirs adjoining houses	5	2.48	4.27
7. Learning CPR skills	2	1.00	1.71
Total	201	100	100

Multiple responses were counted (source: authors).

*Our pond is communal property, and no one can decide alone to install a barrier around the pond; we also cannot bear the expenses alone…*

The two most frequently recommended community-based prevention strategies reported by participants were installing barriers around ponds and other water bodies and increasing awareness of the importance of active child supervision. Participants reported that no organised drowning prevention activities were available in the study area.

The quantitative findings showed that installing barriers around ponds was one of the most frequently recommended community-based prevention strategies. Qualitative interviews provided further insight into why this intervention is difficult to implement, as many ponds are communally owned. In this regard, another participant (60) from Char Teestapara, Chilahati, Debiganj, stated that.

*“Our pond is communal property, and no one can make a decision alone to install a barrier around the pond; we also cannot bear the expenses alone…*”

Except for single houses and their adjoining ponds, all ponds, canals, and rivers are owned communally in the region. In this regard, a participant (33) from Debiganj stated that if a sole proprietorship owned the pond, the owner could install fencing. It was noted that over 99 households live in a *bari*, which consists of several houses located together, with each household owning a single pond.

The second most reported action is to raise awareness among the community or to foster self-awareness; it also involves enhancing understanding of risk. A mother (35) from Taraganj stated, first, we need to be self-aware; then we must also work to create awareness among others. Togetherness and bonding within the community should be harnessed to generate awareness and prevent drowning. The participants also suggested that more knowledgeable people in society could play a role in raising awareness among those who are less informed. A participant (44) expected that community leaders, conscientious individuals, and religious leaders could work together to raise awareness within the community. A participant (19) from Char Teestapara, Chilahati, Debiganj, who lost a 2-year-old child, suggested that they cannot take care of their babies alone; they need to work together as a community to supervise every child and create awareness.

### 3.7 Prevention opportunities

Participants agreed that implementing a package of drowning prevention interventions could help reduce childhood drowning. All households recommended implementing a full package of drowning prevention interventions, including adult supervision and awareness, controlling access to water, providing swimming skills, and pre-schooling initiatives, which could reduce the drowning risk (Table 9). They also agreed to participate in training for water safety, safe rescue, and CPR skills. In response to a question about past help or information they received to prevent child drowning, the participants were unaware of any such initiative. The participants also confirmed that no entities provided training on water safety. Except for one household, all the remaining households (99 per cent) responded positively to the three prevention interventions: (1) sending a 1- to 4-year-old child to any government or private childcare centre; (2) allowing public or private agencies to install a barrier at the entrance of ponds or other types of reservoirs; (3) willingness to send children aged 4 years and above to participate in any swimming training programme organised by any government or private organisation.

**Table 9:** Communities’ acceptance of child drowning prevention interventions.

Prevention interventions	Yes		No	
	Frequency	Percentage	Frequency	Percentage
1. Agreement with the statement that adult supervision and awareness, controlling access to water, providing swimming skills, and pre-schooling initiatives are means for drowning prevention	117	100	0	0
2. Willingness to participate in training in water safety, safe rescue, and CPR skills	117	100	0	0
3. Consenting to send a 1 to 4-year-old child to any government or private childcare centre	116	99.1	1	0.9
4. Public or private agencies installing a barrier at the entrance to your pond or other body of water	116	99.1	1	0.9
5. Willingness to send a child aged 4 and above to participate in any swimming training programme organised by any government or private organisation	116	99.1	1	0.9

Multiple responses were counted (source: authors).

In response to a question about the role the individual would like to undertake as the first initiative to prevent child drowning, we received a variety of responses, including installing barriers at waterbody entrances, establishing childcare facilities, and conducting awareness sessions. Of these, 90 per cent of households preferred installing a barrier at the reservoir’s entrance. Among the remaining 10 per cent of households, 6 and 4 per cent of individuals preferred the establishment of a childcare centre and conducting awareness sessions, respectively.

The participants reported, on many occasions, that parents observed their child and shortly after, they were found dead in a nearby waterbody. A mother (45), from Telipara at Chilahati Union in Debiganj, who lost a 3-year-old child to drowning, reported:

*I saw my son a while ago. Seeing the child was under my surveillance, I engaged in household work. After a while, he was found dead in the pond. If there were a barrier along the pond, this tragic death would have been averted.*

To underscore the necessity of establishing a childcare centre, a father (55) from Majialli at Chilahati, Debiganj, who lost a 3-year-old child, stated that.

*When we were all busy at work, there was a safe place where we could place our child and go to work without worry. As such, we expect GoB to establish a childcare centre for working people who are willing to send their children there.*

Participants perceived that public and private institutions could play an important role in preventing child drowning by raising awareness, fencing waterbodies, filling unnecessary reservoirs, facilitating swimming lessons for children, and establishing a formal childcare centre (Table 10). Where excess and fallow reservoirs and ponds are present, the participants suggested filling them in to mitigate the risk of child drowning. A participant (45) from Masterpara at Chilahati, Debiganj, perceived that partnerships between communities, local administration, and Union Parishad representatives could facilitate the installation of barriers around ponds and strengthen community awareness activities. Participants suggested they cannot afford to do so and lack the legal authority to fill certain types of public reservoirs, which are public property. To do this, intense labour and expenses are required to bring soil from other places. This process also requires public authority involvement and permission.

**Table 10:** Public and private interventions to prevent child drowning.

Responses	Frequency (*n* = 141)	% of responses	% of households
Launching awareness and education programme	64	45.39	54.70
Fencing alongside ponds, canals, and sinks	42	29.79	35.89
Filling sinks, canals, and ponds	14	9.93	11.97
Launching swimming lessons	9	6.38	7.69
Information dissemination about water safety and safe rescue	5	3.55	4.27
Establishing childcare	4	2.84	3.42
CPR training	3	2.13	2.56
Total	141	100	100

Multiple responses were counted (source: authors).

A mother (19) from Char Tistapara at Chilahati, Deviganj, who lost her 2-year-old male child, stated that.

*The Union council can support the fencing of ponds and encourage the community to do the same. Regular awareness activities on child and water safety, fencing and CPR training should be provided to build long-lasting community resilience against childhood drowning. Awareness can also be increased through household visits and seminars organised at local union council levels.*

Quantitative findings indicated that traditional post-rescue practices were widely reported among participants. Qualitative interviews further illustrated that these practices were commonly regarded as appropriate responses to drowning, while participants also recognised important gaps in first-aid knowledge. One caregiver stated,

*The public authority must launch a CPR program to teach people how to treat a drowning child. We do not know how to resuscitate a drowned child. This is the first time I have heard of the term ‘CPR’ through this survey. There should be a public initiative to teach first aid to all.*

Participants described a range of commonly used practices following rescue; however, the present study was not designed to determine whether these practices influenced survival outcomes. Participants also emphasised the need for first aid and CPR training, which could be understood from a participant at Chilahati, Debiganj, who lost a 7-year-old male child:

This quotation highlights participants’ perceived need for formal CPR and first-aid training within the community.

## 4 Discussion

This study provides one of the first mixed-methods investigations of childhood drowning in rural northern Bangladesh, highlighting the interplay of household, behavioral, and environmental factors associated with drowning. The findings showed that most drowning incidents involved children aged 1–4 years, occurred during late morning to early afternoon, and affected predominantly low-income households with limited parental education. Approximately three-quarters of children were reportedly unsupervised immediately before the drowning event, while qualitative findings indicated that caregivers, particularly mothers, were commonly occupied with household chores when incidents occurred. Multivariable analysis identified a greater distance from the household to the drowning site as an independent predictor of fatal drowning, whereas older caregiver age and larger household size were associated with lower odds of fatal outcomes. The study also revealed widespread deficiencies in water safety, swimming, rescue, and cardiopulmonary resuscitation (CPR) knowledge, together with frequent reliance on harmful traditional post-rescue practices. Finally, participants strongly supported community-based prevention measures, particularly installing barriers around ponds and increasing community awareness to improve active child supervision, while also recognising practical challenges to implementing these interventions.

### 4.1 Circumstances surrounding child drowning incidents

At the individual level, participants demonstrated limited knowledge of drowning risk, water safety, CPR, and evidence-based rescue practices. The widespread reliance on traditional post-immersion measures and the limited awareness of formal drowning prevention strategies indicate important knowledge gaps that increase vulnerability to drowning and reduce the likelihood of survival following rescue. These findings are consistent with the social–ecological model, which recognises individual knowledge and beliefs as important determinants of health behavior (23).

Participants frequently perceived poor awareness and inadequate adult supervision as important circumstances associated with childhood drowning, highlighting the need to strengthen community understanding and perception of drowning risk. The findings suggest important gaps in swimming training, water safety awareness, and rescue knowledge among participants from the study communities in northern Bangladesh, while a significant proportion of children lacked formal swimming training. To reduce drowning deaths among children, community-based supervision of young children has proven effective, enhancing active child supervision and opportunities for child development in another region of Bangladesh (12, 18). Most drownings occurred in ponds located within the household environment, suggesting that domestic water bodies remain the principal exposure setting for young children, a finding supported by other literature (35, 36).

### 4.2 Factors associated with fatal drowning

The present study identified distance from the drowning site, caregiver age, and household size as independent factors associated with fatal drowning. The finding that greater distance between households and drowning locations increased the odds of fatal drowning highlights the importance of rapid recognition and timely rescue in drowning outcomes. In rural communities of low- and middle-income countries, children frequently encounter open water bodies located close to households, and delays in reaching and rescuing a drowning child may increase the likelihood of death or severe outcomes. The WHO emphasises that reducing exposure to unsafe water environments, improving community-based supervision, and strengthening timely rescue and resuscitation capacity are essential components of drowning prevention strategies (37, 38).

Caregiver age was also associated with fatal drowning, with older caregivers (≥30 years) showing lower odds of fatal outcomes compared with younger caregivers. This finding may reflect differences in caregiving experience, risk recognition, and the ability to respond effectively during emergencies. Previous evidence indicates that inadequate or ineffective adult supervision is a major contributor to childhood drowning, particularly among children below 5 years of age, and that competent supervision remains a key preventive measure (13, 38). Although supervision status itself was not statistically significant in the adjusted model, unsupervised children showed higher odds of fatal drowning, suggesting that caregiver availability and responsiveness may still play an important role.

The association between larger household size and lower odds of fatal drowning may indicate the potential protective role of increased availability of family members for child monitoring and emergency response. However, household size alone may not guarantee effective supervision, as the quality and attentiveness of supervision are more important than the mere presence of adults (37, 39). The absence of significant associations between parental education and household income after adjustment suggests that socio-economic characteristics may influence drowning vulnerability indirectly through environmental exposure, supervision practices, and access to prevention resources rather than acting as independent determinants. Therefore, drowning prevention programmes should adopt a multisectoral approach combining safer water environments, improved caregiver awareness, community-based childcare, and basic rescue and resuscitation skills, particularly in high-risk rural settings (1).

### 4.3 Community knowledge and perceptions of child drowning

At the interpersonal level, caregivers consistently described inadequate child supervision as a major contributor to drowning incidents. However, these supervision challenges were frequently linked to competing household responsibilities, particularly among mothers. Rather than reflecting simple negligence, these findings suggest structural constraints within caregiving arrangements, highlighting the importance of family-level support systems and alternative childcare options.

A notable finding was the attribution of some drowning deaths to supernatural forces such as jinn or ghosts. Although only a small proportion of participants expressed such beliefs, their presence highlights the influence of cultural interpretations of illness and injury within rural communities. These findings suggest that supernatural beliefs may serve as a coping mechanism for traumatic loss and may also reduce perceived agency for prevention. Similar findings have been reported in other low- and middle-income settings where injuries and sudden deaths are sometimes explained through spiritual or supernatural causes (40). Such beliefs may reduce recognition of modifiable risk factors and delay evidence-based responses to emergencies. Therefore, drowning prevention programmes should incorporate culturally sensitive risk communication strategies that engage community and religious leaders, as well as traditional healers, to address misconceptions while respecting local belief systems. Public health messaging in this context must engage respectfully with local belief systems while promoting evidence-based drowning prevention strategies. Rather than dismissing these beliefs, public health interventions should work within existing cultural frameworks to promote water safety and evidence-based rescue practices.

### 4.4 Post-rescue responses and practices

The widespread use of traditional post-immersion rescue practices represents one of the most concerning findings of this study. Most caregivers lacked knowledge of evidence-based rescue and resuscitation techniques. Instead, they relied on practices such as spinning children over the shoulder, pressing the abdomen, or seeking assistance from traditional healers. Similar observations have been reported in Bangladesh and other low- and middle-income countries where access to first-aid training remains limited (41). The persistence of these practices reflects not only knowledge deficits but also the absence of structured community-based emergency response systems. Given that survival following drowning is highly time-sensitive, delays associated with traditional post-rescue practices may reduce opportunities for timely evidence-based care. These findings underscore the urgent need for community-based CPR and first-aid training programmes integrated into existing health and disaster risk reduction initiatives.

Although drowning prevention efforts often focus on primary immersion events, some cases demonstrate a lack of knowledge that post-rescue first aid can independently contribute to fatal outcomes. Deeply ingrained beliefs about drowning physiology are reflected in the continued use of harmful conventional treatments, such as belly compression and inversion, which impede access to proper medical care. These findings underscore the urgent need for community-based first aid and CPR training, with a particular focus on correcting post-rescue myths that may inadvertently increase mortality risk. This finding highlights a critical and under-recognised dimension of drowning mortality in rural settings: secondary preventable deaths occurring after successful rescue. These cases suggest that limited knowledge of evidence-based post-rescue first aid may have influenced post-rescue management and could have affected outcomes; however, causal inferences cannot be drawn from the present study. The persistence of harmful traditional practices, such as abdominal compression and inversion, reflects deeply embedded misconceptions about drowning physiology and delays access to appropriate medical care. These findings underscore the urgent need for community-based first aid and CPR training, with a particular focus on correcting post-rescue myths that may inadvertently increase mortality risk.

Additionally, the community displayed limited awareness of safe rescue practices, with almost all participants unaware of proper rescue techniques or the need for safety equipment. CPR skills were almost absent, with only a few participants having any knowledge, primarily obtained from informal sources such as social media. Alarmingly, traditional rescue measures, such as spinning children over the shoulders or pressing the abdomen, were widely reported. Participants described several cases in which a child’s death followed these practices despite an initial rescue; however, the study design does not permit conclusions regarding a causal relationship between these practices and mortality. Similar issues were highlighted by Borse et al. (42) in a study underscoring the persistent dangers posed by these harmful and outdated methods. Negligence, unawareness of risks, and misconceptions about drowning prevention further compounded the problem. These findings underscore the urgent need for targeted interventions that address these gaps, such as structured swimming programmes, community education on water safety, CPR training, and the establishment of safe play areas to prevent unsupervised access to water bodies in the region.

A striking finding of this study was the widespread use of traditional post-immersion rescue practices, with almost all participants reporting the use of such measures following drowning incidents. This proportion is considerably higher than that reported by Rahman et al. (21), who found that only 3% of families performed resuscitation, and by Borse et al. (42), who reported that traditional treatment practices were used in 55% of post-immersion cases in Bangladesh. Although direct comparisons should be interpreted cautiously due to differences in study design and settings, the persistence of traditional rescue practices suggests that evidence-based first-aid knowledge remains limited in these rural communities. These findings highlight the need for community-based training on appropriate post-rescue management, including CPR, alongside broader awareness initiatives to promote evidence-based emergency responses following drowning incidents.

### 4.5 Community awareness

At the community level, participants emphasised environmental exposure to ponds, canals, and other open water bodies, as well as the communal ownership of many water resources. Community-level prevention strategies such as awareness campaigns, swimming lessons, childcare centres, and barriers around water bodies were strongly supported by participants, indicating opportunities for locally driven prevention initiatives. At the organisational and policy levels, participants reported a complete absence of drowning prevention interventions, water safety education, CPR training, or structured childcare services. This finding highlights important gaps in institutional support despite substantial evidence demonstrating the effectiveness of drowning prevention interventions in Bangladesh (43, 44). The persistence of similar community recommendations over more than a decade suggests that the primary challenge lies not in identifying effective interventions but in ensuring their sustained implementation and equitable geographic coverage.

The complete absence of formal drowning prevention programmes reported by participants represents a significant public health concern. Despite drowning remaining one of the leading causes of childhood mortality in Bangladesh, households in the study area reported no exposure to water safety education, childcare interventions, swimming programmes, rescue training, or CPR instruction. This finding may reflect geographic inequities in access to drowning prevention initiatives, particularly in northern Bangladesh. While large-scale interventions have demonstrated effectiveness in other regions of the country, their apparent absence in the study area underscores the need to expand proven interventions across a broader geographic area. Policymakers should prioritise integrating drowning prevention into local government planning, primary healthcare outreach, school-based education, and community development programmes to ensure equitable access to prevention services.

### 4.6 Prevention opportunities

An important observation is that many of the prevention measures suggested by participants, including barriers around water bodies, community awareness activities, childcare centres, swimming lessons, and CPR training, closely mirror recommendations reported by Rahman et al. (21) more than 16 years ago. The persistence of these recommendations indicates that, despite longstanding evidence on effective interventions, their implementation and sustainability remain limited. Several factors may contribute to this implementation gap, including limited financial resources, fragmented institutional responsibility, inadequate local government engagement, and the absence of a comprehensive national drowning prevention strategy. The recurrence of identical community recommendations over time and across geographic settings indicates a persistent disconnect between evidence generation and policy implementation in Bangladesh.

Participants emphasised that effective drowning prevention requires collaboration between communities and local authorities, suggesting that partnerships involving community members, local administration, and Union Parishad representatives could facilitate the installation of barriers around ponds and the implementation of community awareness activities. Similar expectations of government support for environmental modifications, particularly pond fencing, were reported by Rahman et al. (21) in Narsingdi. This consistency across different regions of Bangladesh suggests that communities recognise drowning prevention as a shared responsibility requiring coordinated action from both public institutions and local stakeholders. Strengthening community–government partnerships may therefore enhance the planning, implementation, and sustainability of locally appropriate drowning prevention initiatives (46).

Participants proposed a range of community-based strategies to reduce child drowning, including barriers around ponds and other water bodies, increased awareness of active child supervision, swimming lessons, childcare facilities, and CPR training, which emerged as the most frequently recommended interventions. These recommendations are broadly consistent with previous studies conducted in Bangladesh, indicating a continued demand for comprehensive community-based drowning prevention programmes. While environmental modifications such as fencing may be effective for permanent water bodies, their applicability may be limited in rural Bangladesh, where many seasonal or natural water bodies change with climatic conditions (12, 45). Importantly, participants reported the absence of organised drowning prevention activities in the study area, which may reflect limited access to water safety programmes and community awareness initiatives rather than negligence alone. This finding underscores the need for context-specific, multisectoral interventions that combine environmental modifications, community education, supervised childcare, swimming instruction, and first-aid training, while ensuring equitable implementation across underserved regions of Bangladesh (46).

The prevention of child drowning in vulnerable communities requires a collaborative effort that integrates community-led initiatives with institutional support. The findings of this study reflect limited access to community-based prevention programmes and institutional support rather than allowing conclusions regarding negligence at the household, community, or policy level. The findings emphasise the pivotal role of communities in raising awareness, installing barriers around water bodies, and promoting water safety education. While many participants expressed a willingness to contribute to initiatives such as pond fencing and swimming lessons, they perceived that financial constraints, socio-cultural barriers, and the absence of organised local programmes limited their ability to implement community-led drowning prevention measures. Encouragingly, there was unanimous acceptance of child drowning prevention measures, with 99% of households ready to engage in swimming training for children and endorse other preventive actions. However, the absence of formal exposure to drowning prevention initiatives highlights a significant gap in engagement among public and private entities. Participants stressed the urgent need for collaborative interventions, including awareness campaigns, funding for safety infrastructure, and training in CPR and rescue techniques. Addressing these reported challenges may require a coordinated approach where communities, local governments, and NGOs work together to build sustainable, context-specific solutions for child drowning prevention.

## 5 Limitations

Several limitations are notable in this study. Firstly, the findings are confined to two unions in northern Bangladesh; thus, caution is needed in applying them broadly to regions with differing socio-economic and environmental contexts. The study was conducted in two purposively selected unions within northern Bangladesh. Although the selected sites share many socio-economic and environmental characteristics common to rural communities in the region, they may not fully represent the full range of geographic, cultural, and hydrological contexts across northern Bangladesh. Therefore, caution should be exercised when generalising the findings beyond the study locations. Future studies should include a larger number of districts and unions selected through probability-based sampling methods to improve representativeness. The study adopts a cross-sectional design, limiting causal inference; longitudinal studies are essential for understanding causal relationships between socio-demographic factors and drowning incidents. Moreover, self-reported data on socio-economic status, education, and demographics may introduce social desirability bias, potentially skewing results towards socially preferred responses.

Most importantly, the study relies on participants’ recollection of drowning incidents over the past 6 years, which introduces the potential for recall bias. The 6-year retrospective recall period enabled the inclusion of more drowning events but may have reduced the accuracy of participants’ recollections. As a result, these findings should be interpreted as participants’ reported experiences and perceptions rather than as objectively verified accounts of the incidents. Participants may not accurately remember the details of such events, leading to incomplete or inaccurate data. Additionally, drownings that occurred before 2023 may have been omitted from participants’ accounts, further contributing to underreporting. As this study included only drowning-affected households and no comparison group, the reported socio-economic characteristics and knowledge levels should be interpreted as descriptive characteristics of the study population rather than as risk factors or determinants of child drowning.

The inferential analyses should be interpreted with caution. First, the quantitative sample was not designed as a population-representative sample of all children in the study area; rather, it reflects reported drowning-related cases and affected households. Therefore, the analyses estimate associations within observed drowning events rather than population-level incidence or causal risk. Second, several variables contained sparse categories and small cell counts, which limited the reliability of chi-square assumptions and prevented stable multivariable logistic regression estimation. For this reason, exact tests and collapsed-category analyses were used where appropriate, and the multivariable findings were treated as exploratory.

The retrospective study highlights potential recall bias that may affect the accuracy of behavioral and contextual variables related to drowning incidents. Caregiver activities and supervision patterns are often oversimplified by respondents into a single narrative, leading to an exaggerated focus on caregiver negligence. Additionally, as recall accuracy diminishes over time, older cases reported may lack detail, underestimating contextual factors. Consequently, findings on perceived causes should be viewed as subjective rather than definitive determinants. Formal respondent validation (member checking) was not conducted. Future qualitative studies should consider incorporating member checking procedures to strengthen credibility. Future research avenues should prioritise longitudinal studies to monitor drowning trends and evaluate the sustained impacts of preventive interventions. Targeted initiatives, such as childhood swimming lessons and community awareness campaigns, should be implemented and evaluated for their efficacy in reducing drowning incidents. Expanding the study to encompass diverse regions of Bangladesh, including urban and coastal areas, will facilitate the identification of region-specific risk factors and the development of tailored prevention strategies. Detailed assessments of environmental factors, such as water quality and safety features around water bodies, are crucial for designing effective environmental interventions. Qualitative methods, such as in-depth interviews, can illuminate cultural and behavioral influences on drowning incidents, complementing quantitative analyses. Evaluating existing water safety policies and exploring technological solutions, such as mobile apps for real-time incident monitoring, will further enhance prevention efforts.

## 6 Conclusion

The study describes participant-reported circumstances such as lack of child supervision, water safety skills, traditional rescue methods, and the community’s preference for child drowning prevention measures in northern Bangladesh. The findings suggest that low understanding of child drowning risk, unawareness, and a lack of active adult supervision of children due to mothers’ engagement in household chores are the main causes of child drowning deaths in northern Bangladesh. Fatal drowning was independently associated with greater distance from the household, younger caregivers, and smaller household size. These findings suggest that drowning prevention programmes should prioritise households with young caregivers and limited family support, while improving supervision around water bodies located farther from homes. Community-based childcare, targeted caregiver education, and environmental modifications should be integrated into local drowning prevention policies. Overall, participants had limited knowledge of water safety, swimming skills, the prevalence of child drowning, and prevention measures. Neither the community, private, nor non-governmental organisations (NGOs) provided any drowning prevention interventions. Lacking knowledge of CPR, 94% of respondents performed malpractice during the post-immersion period, resulting in child deaths. Although the community has limited knowledge about drowning prevention measures, when exposed to the full package of drowning prevention interventions, the participants agreed to accept it in their local context. At the household level, the participants prefer to install fencing around pond walkways and other types of reservoirs. The participants anticipate the support and involvement of both public and private organisations in raising awareness, fencing and filling ponds, and conducting swimming lessons. This study has found the need for public policy to implement preventive measures, specifically filling certain types of reservoirs and fencing communal ponds. The findings may be of interest to relevant public and private organisations seeking to raise awareness and build capacity in the region. This study suggests that it will require synergies among individuals, households, communities, local councils, and the civil administration to prevent child drowning and promote child development in Bangladesh. At the national level, the study recommends the formulation and implementation of a comprehensive national water safety policy. Viewed through the social–ecological model, childhood drowning in northern Bangladesh emerges as a multidimensional public health problem shaped by interacting individual, interpersonal, community, and policy-level factors. Effective prevention will therefore require integrated interventions that simultaneously improve caregiver awareness, strengthen child supervision, address environmental hazards, enhance community capacity for rescue and resuscitation, and expand institutional support for drowning prevention programmes.

## Data Availability

The original contributions presented in the study are included in the article/supplementary material, further inquiries can be directed to the corresponding author.
